# Schizophrenia and subsequent neighborhood deprivation: revisiting the social drift hypothesis using population, twin and molecular genetic data

**DOI:** 10.1038/tp.2016.62

**Published:** 2016-05-03

**Authors:** A Sariaslan, S Fazel, B M D'Onofrio, N Långström, H Larsson, S E Bergen, R Kuja-Halkola, P Lichtenstein

**Affiliations:** 1 Department of Psychiatry, University of Oxford, Warneford Hospital, Oxford, UK; 2 Department of Medical Epidemiology and Biostatistics, Karolinska Institutet, Stockholm, Sweden; 3 Department of Psychological and Brain Sciences, Indiana University, Bloomington, IN, USA; 4 Department of Medical Sciences, Örebro University, Örebro, Sweden

## Abstract

Neighborhood influences in the etiology of schizophrenia have been emphasized in a number of systematic reviews, but causality remains uncertain. To test the social drift hypothesis, we used three complementary genetically informed Swedish cohorts. First, we used nationwide Swedish data on approximately 760 000 full- and half-sibling pairs born between 1951 and 1974 and quantitative genetic models to study genetic and environmental influences on the overlap between schizophrenia in young adulthood and subsequent residence in socioeconomically deprived neighborhoods. Schizophrenia diagnoses were ascertained using the National Patient Registry. Second, we tested the overlap between childhood psychotic experiences and neighborhood deprivation in early adulthood in the longitudinal Twin Study of Child and Adolescent Development (TCHAD; *n*=2960). Third, we investigated to what extent polygenic risk scores for schizophrenia predicted residence in deprived neighborhoods during late adulthood using the TwinGene sample (*n*=6796). Sibling data suggested that living in deprived neighborhoods was substantially heritable; 65% (95% confidence interval (95% CI): 60–71%) of the variance was attributed to genetic influences. Although the correlation between schizophrenia and neighborhood deprivation was moderate in magnitude (*r*=0.22; 95% CI: 0.20–0.24), it was entirely explained by genetic influences. We replicated these findings in the TCHAD sample. Moreover, the association between polygenic risk for schizophrenia and neighborhood deprivation was statistically significant (*R*^2^=0.15%, *P*=0.002). Our findings are primarily consistent with a genetic selection interpretation where genetic liability for schizophrenia also predicts subsequent residence in socioeconomically deprived neighborhoods. Previous studies may have overemphasized the relative importance of environmental influences in the social drift of schizophrenia patients. Clinical and policy interventions will therefore benefit from the future identification of potentially causal pathways between different dimensions of cognitive functions and socioeconomic trajectories derived from studies adopting family-based research designs.

## Introduction

The pioneering work of Faris and Dunham^1^ in the late 1930s found increased incidence of schizophrenia in socially disorganized residential areas characterized by high rates of poverty, marital instability, residential mobility, and ethnic heterogeneity. Despite considerable efforts to understand the nature of this ecological association, its potential causal mechanisms (both in terms of the direction of the effects as well as the underlying etiology) remain largely uncertain.

Proponents of the social causation interpretation have proposed a number of hypotheses postulating that the development of schizophrenia results from early exposure to adverse neighborhood stressors, accumulated over time, particularly in individuals with genetic liabilities.^2, 3, 4, 5^ In contrast, the drift interpretation suggests that the association goes in the opposite direction; namely that psychotic patients tend to gradually undergo downward social mobility (or to ‘drift') into deprived neighborhoods following employment difficulties associated with experiencing psychotic-like symptoms and declining cognitive functioning.^6, 7^

Systematic reviews have generally claimed support for the social causation interpretation.^8, 9^ However, the evidence is limited and inconclusive; most of the findings are either based on small and selected clinical samples, ecological study designs with inadequate adjustment for individual-level confounders or population-based studies with genetically uninformed designs. Large-scale, eco-epidemiological studies that simultaneously account for individual, familial and social contextual determinants are therefore integral in clarifying underlying mechanisms.^10, 11^ The only genetically informed study using nationwide longitudinal registry data failed to support the causal interpretation; the authors concluded that familial confounding fully explained the impact of neighborhood characteristics (for example, deprivation and urbanicity), measured from birth up to adolescence, on later schizophrenia.^12^

Much less is known about the magnitude and etiology of drift mechanisms in patients with schizophrenia. Quantitative and molecular genetic studies have consistently found that indicators of socioeconomic status are considerably influenced by genetic influences.^13^ Notably, a recent Scottish twin study^14^ estimated the heritability of deprived neighborhood residence to 71% (95% confidence interval: 69–73%), which is similar in magnitude to that of schizophrenia.^15^ What remains unknown is to what degree the genetic influences that contribute to the liability of schizophrenia overlap with those of neighborhood deprivation. In contrast to studies on poor social functioning outcomes in individuals with schizophrenia that tend to focus on environmental explanations (for example, marked changes to social policy and socioeconomic disparities),^16, 17, 18, 19, 20^ a shared genetic etiology between schizophrenia and socioeconomic deprivation would imply that such associations are, at least partly, due to genetic confounding.

We adopted multiple genetically informed designs to address the following aims:

1. To estimate the contributions of genetic and environmental influences on adulthood deprived neighborhood residence.
2. To estimate such influences on the overlap between schizophrenia and subsequent residence in deprived neighborhoods.
3. To explore the association between subclinical measures of psychotic experiences during childhood and adolescence with neighborhood deprivation during early adulthood.
4. To examine whether polygenic risk scores for schizophrenia predict neighborhood deprivation in late adulthood.

## Materials and methods

### The sibling study

#### Swedish nationwide registries

Statistics Sweden maintains all nationwide Swedish longitudinal registries with routinely gathered governmental data. Individual data can be linked across registers via a unique civic registration number assigned to all Swedish residents at birth or upon immigration. To ensure confidentiality, we were granted access to anonymized data after approval from the Regional Research Ethics Committee at Karolinska Institutet.

We used the Total Population Register to identify all individuals born in Sweden between 1951 and 1974 and linked their data with the Multi-Generation Register to identify all biological full and half siblings in the sample and generate family pedigree structures. Censoring information on mortality and migration dates was obtained from the Cause of Death and Migration Registries, respectively. We identified individuals with hospital discharges and associated schizophrenia diagnoses using the National Patient Register that encompasses data on psychiatric inpatient care since 1973 (ICD-8, -9 and -10) as well as specialist (non-general practitioner) outpatient care since 2001 (ICD-10). The Small Area Marketing Statistics Register provided data on annual neighborhood residence between 1982 and 2009. Neighborhood characteristics data were aggregated from the Total Population Register, census registries, as well as the National Crime Register, which includes all criminal convictions in lower general court in Sweden since 1973.

#### Subjects

Between 1951 and 1974, a total of 2 628 631 live births that could be linked to two biological parents were registered in Sweden. We excluded individuals who died (*n*=41 440) or emigrated (*n*=163 868) before age 35 years to have sufficient follow-up time. Further, we excluded those who could not be assigned to residential areas between ages 31 to 35 (*n*=37 315) resulting in a sample of 90.8 percent (*n*=2 386 008) of the original cohort. From this population sample, we subsequently identified all biological full siblings (*n*=1 615 719), maternal half siblings (*n*=232 720) and paternal half siblings (*n*=279 758). In families with multiple offspring, we selected the oldest two siblings born within 5 years from one another to reduce bias due to violation of the equal environment assumption for siblings in our analyses. The final sample included 759 536 sibling pairs (592 009 full-sibling pairs, 68 684 maternal half-sibling pairs and 82 913 paternal half-sibling pairs).

### Measures

#### Schizophrenia

We defined study participants as having schizophrenia if they had been hospitalized with any diagnosis of schizophrenia (ICD-8/9: 295; ICD-10: F20) on at least two separate occasions, irrespective of any psychiatric comorbidity, before age 31 years. Although single-episode hospitalizations of schizophrenia have been validated in the Swedish Patient Registry;^21^ this stricter definition is commonly used to increase diagnostic precision by minimizing the risk of obtaining false-positive cases.^15^

#### Neighborhood deprivation

We defined neighborhoods according to the Small Area Marketing Statistics classification system that delineates very small and socioeconomically homogenous residential areas,^22, 23^ Annual neighborhood deprivation scores were generated on the basis of the following aggregated indicators; proportion of residents aged 25–64 years with no secondary school qualifications, immigrant status, divorced and the neighborhood crime rate.^12^ Despite Sweden's comprehensive welfare policies, previous studies suggest stark socioeconomic differences^22, 24^ and the occurrence of psychotic disorders has been found to be strongly nonlinearly distributed across neighborhoods with the highest concentrations being observed in the most deprived areas.^12, 25^ To account for such nonlinear effects, we used a binary measure indicating whether the study participant had ever resided in a deprived neighborhood, defined as the 95th percentile of the continuous distribution, between ages 31 and 35. A total of 260 957 study participants, corresponding to approximately 11 percent of the sample, fulfilled this criterion.

### Analyses

#### Sibling models

In our initial analyses, we estimated odds ratios (ORs) for living in a deprived neighborhood in siblings of a proband with schizophrenia compared with sibling of an individual without schizophrenia using the binary logistic regression model. For genetically influenced traits, larger ORs for full rather than half siblings are expected given that they share on average 50 and 25 percent of their co-segregating genes, respectively. Similarly, for traits to be influenced by shared childhood environmental factors, we expected the ORs for maternal half siblings to be larger than those for paternal half siblings owing to the assumption that the former share their childhood environment to a substantially greater extent.^26^

#### Quantitative genetic sibling models

We subsequently fitted univariate, quantitative genetic structural equation models^27^ that explicitly estimate the relative importance of three sources of variation in schizophrenia and neighborhood deprivation; additive genetic effects, shared environmental effects and unique environmental effects, the latter of which also includes measurement error. We estimated these sources of variation by constraining sibling correlations in the variance components to their expected values; additive genetic correlations between full siblings was set to 0.5 and half siblings to 0.25, respectively, while the shared environmental correlation was set to 1.0 for full siblings, 0.83 for maternal half siblings and 0.03 for paternal half siblings. The latter two assumptions were empirically chosen on the basis of a study demonstrating that 83 percent of maternal half siblings and 3 percent of paternal half siblings were registered to live in the same household in the 1960–1990 Swedish national censuses.^28^ We accounted for potential gender and cohort effects by adjusting for sex and birth year as covariates in all the models.

By implementing the Cholesky decomposition method,^29^ we estimated the bivariate correlation between schizophrenia and neighborhood deprivation and stratified the correlation by genetic and environmental influences. We additionally specified a liability-threshold model to account for the binary nature of our phenotypes^30^ but relaxed the equal thresholds assumption because we observed differences in the prevalence of the phenotypes between full and half siblings. All the models were fitted in R/OpenMx 1.3.^31^

### The twin study

#### Subjects

The TCHAD (Twin Study of Child and Adolescent Development)^32^ is a population-based longitudinal twin study including all twins born in Sweden between May 1985 and December 1986 who were alive and Swedish residents at the end of 1994 (*n*=2960). The study participants and their parents were surveyed via mailed questionnaires at three different time points (1994, 1999 and 2002). We excluded study participants whose parents had decided not to participate and those who lacked information on their residential areas in adulthood (*n*=240). Although participation rates were substantially lower in ethnically heterogeneous residential areas, the attrition rates at follow-up did not vary by reported psychotic symptoms.^33^ The final sample included 1355 twin pairs, distributed over 533 monozygotic twin pairs and 822 dizygotic twin pairs. The determination of zygosity was based on a validated algorithm derived from questionnaire data measuring the physical similarities between the twins as well as the extent to which others tend to confuse them for one another.^32^

### Measures

#### Psychotic experiences

We classified children whose parents reported that their child had experienced auditory hallucinations according to the Child Behavior Checklist anytime throughout the studied period as having had psychotic experiences.^34^ Measures of auditory hallucinations, especially when reported by parents,^35^ have been found to strongly predict psychotic experiences in general.^36^

#### Neighborhood deprivation

Neighborhood deprivation was measured during 2 years (2008 and 2009) for the TCHAD sample and using a wider definition (75th percentile) owing to the small sample size and relatively fewer participants from the most deprived neighborhoods.

#### Analyses

Quantitative genetic twin models

We fitted quantitative genetic models to the TCHAD data equivalent to those used for siblings as described above. Genetic correlations between monozygotic and dizygotic twins were set to 1.0 and 0.5, respectively, and we assumed that both groups shared childhood environments with their co-twins.

### Polygenic risk scores

#### Subjects and genotyping

The TwinGene study includes a total of 22 390 twins born 1958 or earlier, invited to participate between 2004 and 2008. Overall, 12 614 twins (56.3%) consented to participate and donated blood samples, which were genotyped using the Illumina OmniExpress chip containing probes for single-nucleotide polymorphisms (SNPs). The details on the data collection procedures have been described elsewhere.^37^ One twin from each pair was randomly selected for inclusion to yield a data set of 6796 unrelated individuals, avoiding potential biases from analyses of genetically correlated subjects.

### Measures

#### Neighborhood deprivation

Owing to the TwinGene study design, the study participants were increasingly economically affluent and healthy as compared with population controls. Therefore, we decided to study the relative differences in their exposure to neighborhood deprivation by averaging their continuous deprivation scores between 1999 and 2004.

### Analysis

The discovery SNP set was generated from the results of the genome-wide association study of schizophrenia from the Psychiatric Genomics Consortium^38^ and comprised 97 046 SNPs in relative linkage equilibrium. The weightings for each SNP based on the strength of its association in the discovery genome-wide association study were applied to the target sample, TwinGene, to calculate aggregated polygenic risk scores for each subject using PLINK v1.07.^39^ The scores were generated for *P*-value thresholds from 0.01 to 1. We fitted linear regression models to assess the proportion of variance in neighborhood deprivation attributed to the polygenic risk scores, incorporating 10 principal components accounting for population substructure and estimated the *R*^2^ by subtracting the effects due to the covariates alone.

## Results

We estimated that 0.4% of the full-sibling pairs had at least one occurrence of schizophrenia while the equivalent estimate in half siblings was 0.6% (Table 1). Moreover, we found that sibling pairs who had been affected by schizophrenia were twice as likely to live in deprived neighborhoods between ages 31 and 35 than population controls (24 vs 12%). Similar results were found for those affected by psychotic experiences in the twin study.

In agreement with previous studies,^15^ we found a high familial recurrence risk for schizophrenia (Supplementary eTable 1). Similarly, we found that living in deprived neighborhoods in adulthood also tended to run in families (OR=2.10–3.24; eTable 2). The likelihood of living in deprived neighborhoods was increased for individuals diagnosed with schizophrenia compared with unrelated controls (OR=2.88–3.70; Table 2). The corresponding OR for a full sibling to a proband with schizophrenia was also substantially increased (OR=1.90). Comparisons across sibling types demonstrated that the likelihood of residing in a deprived neighborhood was substantially higher among full siblings than half siblings, suggesting the presence of genetic influences. Commensurate results were found for psychotic experiences in the twin study, such that the odds to live in a deprived neighborhood for a monozygotic twin to a proband with psychotic experiences was substantially increased (OR=7.03) as compared with a dizygotic twin (OR=1.23).

The univariate quantitative genetic models are presented in Table 3. We found that neighborhood deprivation in adult Swedish siblings, between ages 31 and 35, was substantially heritable with additive genetic influences accounting for 65% of the variance. Further, 3% of the variance was attributed to shared childhood environmental influences. In the twin study, when twins in the age range 23 to 24 were assessed for neighborhood deprivation, the heritability estimate was found to be reduced by approximately a third (41%). Additive genetic influences substantially explained the differences in both schizophrenia and psychotic experiences (73% and 90%, respectively), whereas we did not find any shared environmental influences.

**Figure 1:**
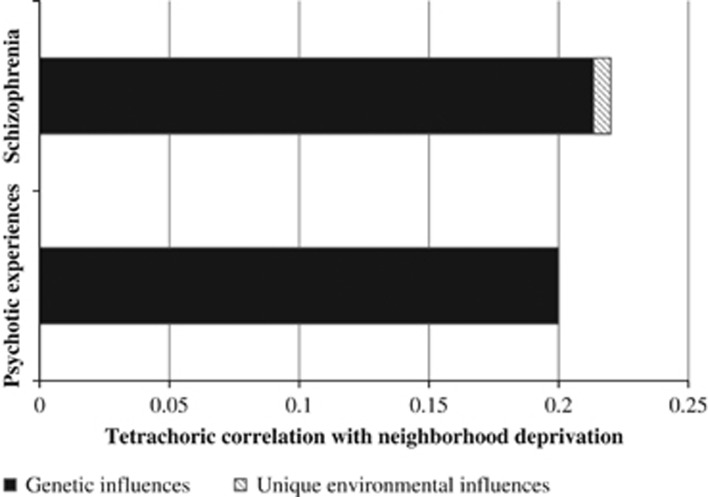
Tetrachoric correlations between schizophrenia, psychotic experiences and neighborhood deprivation, stratified by genetic and environmental influences. All models adjust for sex and birth year. The tetrachoric correlation coefficient is estimated for two binary variables under the assumption of a latent bivariate normal distribution. All models adjust for sex and birth year. The tetrachoric correlation coefficient is estimated for two binary variables under the assumption of a latent bivariate normal distribution.

The bivariate quantitative genetic models (Figure 1) revealed a moderately sized correlation between schizophrenia and neighborhood deprivation (*r*=0.22; 95% confidence interval: 0.20–0.24), which was replicated in the twin study (*r*=0.20; 0.04–0.36). The partitioning of the phenotypic correlations into genetic and environmental influences demonstrated that genetic influences almost entirely accounted for these correlations. The unique environmental influences were not significantly different from zero (*P*_schizophrenia_=0.58; *P*_psychotic experiences_=0.29). Complete model parameters are presented in Supplementary eTable 3.

Last, we found an association between polygenic risk scores for schizophrenia and living in deprived neighborhoods in mid-to-late adulthood across the *P*-value thresholds for the SNPs (Table 4). All of the associations were statistically significant from zero (*P*<0.05) and increasing as a function of the thresholds. The polygenic risk scores for schizophrenia explained approximately 0.15% of the variance in neighborhood deprivation when using all SNPs.

## Discussion

We investigated the influences of schizophrenia and psychotic experiences on subsequent residence in deprived neighborhoods. We found that living in deprived neighborhoods in adulthood was a substantially heritable trait with genetic influences accounting for 65% of the observed variation. Consistent with recent systematic reviews,^8, 9^ we found that individuals diagnosed with schizophrenia, as well as children with subclinical psychotic experiences, were more likely to live in deprived neighborhoods as compared with population controls. However, in contrast to these reviews, we found limited support for the interpretation that environmental factors causes the social drift into deprived neighborhoods because the associations were fully confounded by genetic influences. Complementary analyses using molecular genetic data revealed, for the first time to our knowledge, that polygenic risk for schizophrenia in nonclinical participants predicted living in deprived neighborhoods in late adulthood.

Consistent with a recent Scottish twin study,^14^ we found that genetic influences seem to explain 65 percent of the variance in adulthood residence in deprived neighborhoods measured over a period of 5 years. Together, these findings indicate that there is genetic liability that increases the likelihood of living in deprived neighborhoods in the entire population and not only among individuals diagnosed with schizophrenia and their relatives. It is important to note, however, that environmental influences, and in particular those that are not shared within a family, remain important and account for approximately a third of the variation in deprived neighborhood residence.

Our next three aims focused specifically on understanding the underlying mechanisms that might explain why individuals with psychotic disorders tend to live in deprived neighborhoods. We found that the association between schizophrenia and adulthood neighborhood deprivation, albeit moderate in magnitude, was fully explained by shared genetic influences. In other words, the excess likelihood of adulthood residence in deprived neighborhoods in schizophrenia patients was explained by genetic factors that also increased their liability to be diagnosed with schizophrenia.^40^ Our twin data suggested a similar mechanism for the likelihood of early adulthood residence in deprived neighborhoods among children experiencing auditory hallucinations. Importantly, this link was also apparent on the molecular level when we observed statistically significant associations between polygenic risk scores for schizophrenia among non-patients and neighborhood deprivation in late adulthood. The shared genetic influences likely target a broader spectrum of problems, including psychotic symptoms, cognitive impairments and language difficulties that are more common in families with individuals with non-affective psychotic disorders.^41, 42^ These problems have previously been linked to poor vocational outcomes (for example, failure to maintain stable employment, low earnings and so on), particularly in patients diagnosed with schizophrenia,^43^ but our findings suggest that the elevated rates of these problems also extend to residents of deprived neighborhoods.

Our results should be interpreted in light of some methodological limitations. First, we relied on national patient registry data for the ascertainment of schizophrenia cases and we adopted a narrow definition of the disorder to achieve a high level of specificity. Sensitivity tests using a broader measure of non-affective psychosis (Supplementary eTable 4) was nevertheless commensurate with the presented findings. Second, given that the National Patient Register provides data starting from 1973, which implies that the oldest cohort members were 22 years of age at the time of its inception, we may have misclassified schizophrenia exposure for some of the older individuals. The extent of this bias is likely minimal, however, as we found that the prevalence rate of schizophrenia was higher, albeit marginally, in cohorts born between 1951 and 1958 (0.29% 95% confidence interval: 0.27–0.30%) than among their younger peers who were born between 1959 and 1967 (0.25% 95% confidence interval: 0.24–0.26%). Third, we used broader neighborhood deprivation measures in the included twin studies due to a combination of power and sample selection issues as described above. However, sensitivity tests using broader deprivation measures in the sibling study supported our conclusions (Supplementary eTable 5). Fourth, we were unable to assess neighborhood exposure before the onset of schizophrenia in the sibling study owing to the lack of neighborhood data before 1982. However, our approach enabled us to have follow-up data spanning almost three decades. Moreover, the complementary twin study included exposure data preceding adulthood neighborhood residence (that is, where the children were still living in their parents' home when they reported the hallucinations) and replicated the findings of the sibling study. Fifth, we estimated substantially smaller, albeit statistically significant, effects using the polygenic risk scoring method as compared with the quantitative genetic models. This was expected, however, because GWA studies only account for common variants.^44^

Last, quantitative genetic models involve a number of assumptions, including, but not limited to; no assortative mating, equal exposure across sibling types and a constant shared environmental correlation for each sibling type.^11^ Although assortative mating in schizophrenia is established,^45^ a violation of this assumption will likely contribute to a downward bias of the genetic influences.^46^ Half siblings tend to experience higher prevalence of both phenotypes, which is why we allowed for the estimation of different thresholds across sibling types. The specification of a constant shared environmental correlation for Swedish half siblings may be problematic as the correlation has been shown to vary across birth cohorts (for example, the proportion of half siblings that share their environments have increased in younger cohorts) as well as with age (for example, half siblings tend to share their environments to a lesser extent as they transition from early childhood to adolescence), particularly in paternal half siblings.^47^ We conducted, therefore, two sets of complementary sensitivity analyses to test the stability of the presented parameter estimates. First, we specified a number of alternative and plausible shared environmental correlations for the half siblings (Supplementary eTable 6) but only observed negligible differences. Second, we cross-linked the baseline population with the data from the Swedish Twin Registry, which allowed us to identify all monozygotic twins (*n*=8830) and dizygotic twins (*n*=23 030) in the population sample. We subsequently re-fitted the quantitative genetic models on the sample of twins and full siblings. The results matched the presented findings; a moderately sized correlation (*r*=0.22; 0.19–0.26) that was entirely attributed to common genetic influences (for example, the unique environmental contributions were not significantly different from zero; *P*=0.22).

## Conclusions

Our findings suggest that the association between schizophrenia and later residence in deprived neighborhoods is consistent with a genetic selection interpretation where the overlap between the phenotypes is primarily explained by common genetic influences. Considering recent developments within psychiatric genetics to incorporate environmental risk factors into molecular genetic investigations of psychiatric disorders (for example, exploring gene–environment interactions), the findings of this study highlight the importance of combining such endeavors with family-based research designs to further our understanding of the underlying causal mechanisms.^11, 48, 49^ The development of clinical and policy interventions will, therefore, benefit from future identification of potential pathways between different dimensions of cognitive functions and socioeconomic trajectories.

## Figures and Tables

**Table 1:** Percentage of sibling and twin pairs with and without psychotic disorders as well as the proportion of pairs where at least one sibling had ever lived in a deprived neighborhood in adulthood

	*Sibling study*			*Twin study*	
	*Not affected by schizophrenia*	*At least one sibling affected by schizophrenia*		*Not affected by psychotic experiences*	*At least one twin affected by psychotic experiences*
Full-sibling pairs	99.6% (99.6% 99.6%)	0.4% (0.4% 0.5%)	MZ twin pairs	97.6% (96.3% 98.9%)	2.4% (1.1% 3.8%)
Maternal half-sibling pairs	99.4% (99.4% 99.5%)	0.6% (0.5% 0.6%)	DZ twin pairs	96.8% (95.5% 98.0%)	3.4% (2.0% 4.5%)
Paternal half-sibling pairs	99.4% (99.4% 99.5%)	0.6% (0.5% 0.6%)			
Proportion ever lived in a deprived neighborhood between ages 31 and 35 years	0.12 (0.11; 0.12)	0.24 (0.23; 0.26)	Proportion ever lived in a deprived neighborhood between ages 23 and 24 years	0.35 (0.32; 0.37)	0.50 (0.35; 0.65)

Abbreviations: DZ twin, dizygotic twin; MZ twin, monozygotic twin.

Neighborhoods in the 95th and 75th percentile of standardized deprivation scores measured annually based on the proportion of residents with low educational attainment, divorced, immigrants and the neighborhood crime rate were classified as being deprived in the sibling and twin study, respectively.

**Table 2:** Likelihood for neighborhood deprivation in individuals with schizophrenia and psychotic experiences as well as for siblings and twins of affected individuals

*Sibling study*			*Twin study*		
*Relation to proband*	*Risk for neighborhood deprivation in individuals with schizophrenia*	*Risk for neighborhood deprivation in siblings to proband with schizophrenia*	*Relation to proband*	*Risk for neighborhood deprivation in individuals with psychotic experiences*	*Risk for neighborhood deprivation in twins to proband with psychotic experiences*
	**OR (95% confidence intervals)**	**OR (95% confidence intervals)**		**OR (95% confidence intervals)**	**OR (95% confidence intervals)**
Full siblings	3.70 (2.29; 4.16)	1.90 (1.65; 2.19)	MZ twins	6.91 (1.45; 32.87)	7.03 (1.48; 33.46)
Maternal half siblings	2.88 (2.19; 3.78)	1.62 (1.16; 2.26)	DZ twins	0.89 (0.30; 2.58)	1.23 (0.44; 3.43)
Paternal half siblings	3.09 (2.38; 4.01)	1.28 (0.92; 1.77)			

Abbreviations: DZ twin, dizygotic twin; MZ twin, monozygotic twin; OR, odds ratios.

Neighborhoods in the 95th and 75th percentile of standardized deprivation scores measured annually based on the proportion of residents with low educational attainment, divorced, immigrants and the neighborhood crime rate were classified as being deprived in the sibling and twin study, respectively.

**Table 3:** Variance components for additive genetic, shared childhood environmental and unique environmental influences derived from univariate quantitative genetic models for neighborhood deprivation, schizophrenia and psychotic experiences

	*Additive genetic influences*	*Shared environmental influences*	*Unique environmental influences*
*Sibling study*			
Neighborhood deprivation	0.65 (0.60; 0.71)	0.03 (0.00; 0.05)	0.32 (0.29; 0.35)
Schizophrenia	0.73 (0.65; 0.81)	0.00 (0.00; 0.00)	0.27 (0.19; 0.35)
			
*Twin study*			
Neighborhood deprivation	0.41 (0.15; 0.67)	0.26 (0.05; 0.47)	0.32 (0.24; 0.42)
Psychotic experiences	0.90 (0.59; 0.98)	0.00 (0.00; 0.00)	0.10 (0.01; 0.31)

All the models adjust for sex and birth year.

Confidence intervals were derived using the delta method.

**Table 4:** Polygenic risk scores for schizophrenia across *P*-value thresholds (pT) predicting neighborhood deprivation in late adulthood in the TwinGene sample based on linear regression models

*pT*	R^*2*^	P*-value*
0.01	0.0008	0.019
0.05	0.0010	0.008
0.10	0.0011	0.007
0.20	0.0011	0.005
0.30	0.0012	0.005
0.40	0.0012	0.005
0.50	0.0014	0.002
1.00	0.0015	0.002
