# Supplementary material for: Schizophrenia and subsequent neighborhood deprivation: revisiting the social drift hypothesis using population, twin and molecular genetic data
Source: Transl Psychiatry. 2016 May 3;6(5):e796–. doi: 10.1038/tp.2016.62 (PMC5070045; doi:10.1038/tp.2016.62)
Supplement: Supplementary Information [file tp201662x1.docx]

**Supplementary information: Recurrence odds ratios**

We fitted binary logistic regression models to estimate recurrence odds ratios, and corresponding 95% confidence intervals, predicting schizophrenia (eTable 1) and neighborhood deprivation (eTable 2).

The recurrence odds ratio is interpreted as the odds to develop the disorder (schizophrenia) in an individual who has a sibling that has been diagnosed with the disorder, compared to an individual whose sibling has not been diagnosed with the disorder (schizophrenia).

We present the findings stratified across sibling types (full-siblings, maternal and paternal half-siblings) to descriptively explore whether the associations are due to genetic (e.g. full-siblings experience higher odds than half-siblings) and/or environmental influences (e.g. maternal half-siblings experience higher odds than paternal half-siblings).

**eTable 1.** Recurrence odds ratios for schizophrenia and psychotic experiences with 95% confidence intervals.

| **Sibling study** | |  | **Twin study** | |
| --- | --- | --- | --- | --- |
| **Relation to proband** | **Schizophrenia** |  | **Relation to proband** | **Psychotic experiences** |
| Full-siblings | 15.2 [11.2; 20.8] |  | MZ twins | 242 [38; 1560] |
| Maternal half-siblings | 8.5 [3.5; 20.9] |  | DZ twins | 5.9 [0.7; 51.0] |
| Paternal half-siblings | 10.9 [5.1; 23.3] |  |  |  |

**eTable 2.** Recurrence odds ratios for neighborhood deprivation with 95% confidence intervals.

| **Sibling study** | |  | **Twin study** | |
| --- | --- | --- | --- | --- |
| **Relation to proband** | **Neighborhood deprivation** |  | **Relation to proband** | **Neighborhood deprivation** |
| Full-siblings | 3.24 [3.17; 3.31] |  | MZ twins | 8.15 [5.47; 12.16] |
| Maternal half-siblings | 2.52 [2.40; 2.64] |  | DZ twins | 3.77 [2.77; 5.12] |
| Paternal half-siblings | 2.10 [2.01; 2.20] |  |  |  |

**eTable 3.** Model parameters derived from bivariate quantitative genetic models. Confidence intervals were derived using the delta method. Variance components for additive genetic, shared childhood environmental and unique environmental influences are denoted by a, c, and e, respectively.

|  | **Schizophrenia** | **Psychotic experiences** |
| --- | --- | --- |
| **Disorder** |  |  |
| a11 | 0.88 [0.88; 0.88] | 0.96 [0.86; 1.05] |
| c11 | 0* | 0* |
| e11 | -0.47 [-0.55; -0.40] | 0.29 [0.09; 0.49] |
| **Overlap** |  |  |
| a12 | 0.24 [0.24; 0.24] | 0.29 [0.03; 0.54] |
| c12 | 0* | 0* |
| e12 | -0.01 [-0.08; 0.05] | -0.25 [-0.85; 0.34] |
| **Neighborhood deprivation** |  |  |
| a22 | 0.77 [0.73; 0.82] | 0.78 [0.66; 0.91] |
| c22 | 0.15 [0.08; 0.22] | 0.55 [0.36; 0.73] |
| e22 | -0.56 [-0.58; -0.54] | 0.53 [0.23; 0.83] |

**Notes:** We fixed the shared environmental estimates for schizophrenia and psychotic experiences to zero following the results of the univariate models to facilitate model convergence. The figures 11, 22 and 12 denote influences on the disorder, neighborhood deprivation and the overlap between them, respectively. Both models adjust for sex and birth year.

**eTable 4.** Sensitivity analyses. Univariate and bivariate quantitative genetic sibling models testing broader non-affective psychotic disorder as an alternative phenotype. Confidence intervals were derived using the delta method.

|  | **Univariate models** | | |  | **Bivariate models** | | |
| --- | --- | --- | --- | --- | --- | --- | --- |
|  | **Additive genetic influences** | **Shared environmental influences** | **Unique environmental influences** |  | **Phenotypic**  **correlation** | **Proportion of correlation due to genetic influences** | **Proportion of correlation due to unique environmental influences** |
| Schizophrenia  2+ episodes  (Reference) | 0.73 [0.65; 0.81] | 0.00 [0.00; 0.00] | 0.27 [0.19; 0.35] |  | 0.22 [0.20; 0.24] | 0.97 [0.87; 1.07] | 0.03 [-0.07; 0.13] |
| Non-affective psychosis  1+ episodes | 0.68 [0.63; 0.72] | 0.00 [0.00; 0.00] | 0.32 [0.28; 0.37] |  | 0.21 [0.20; 0.22] | 1.08 [1.05; 1.11] | -0.08 [-0.11; 0.05] |

Note: All models adjust for sex and birth year. Non-affective psychosis includes the following diagnosis codes: 295-299 (ICD-8); 295-288 (ICD-9) and F20-F29 (ICD-10).

**eTable 5.** Sensitivity analyses. Univariate and bivariate quantitative genetic sibling models testing broader definitions of neighborhood deprivation. Confidence intervals were derived using the delta method.

|  | **Univariate models** | | |  | **Bivariate models** | | |
| --- | --- | --- | --- | --- | --- | --- | --- |
|  | **Additive genetic influences** | **Shared environmental influences** | **Unique environmental influences** |  | **Phenotypic**  **correlation** | **Proportion of correlation due to genetic influences** | **Proportion of correlation due to unique environmental influences** |
| 95^th^ percentile (reference) | 0.65 [0.60; 0.71] | 0.03 [0.00; 0.05] | 0.32 [0.29; 0.35] |  | 0.22 [0.20; 0.24] | 0.97 [0.87; 1.07] | 0.03 [-0.07; 0.13] |
| 90^th^ percentile | 0.60 [0.56; 0.64] | 0.01 [-0.01; 0.03] | 0.39 [0.36; 0.41] |  | 0.22 [0.22; 0.23] | 0.81 [0.78; 0.83] | 0.19 [0.17; 0.22] |
| 85^th^ percentile | 0.53 [0.52; 0.53] | 0.02 [0.02; 0.03] | 0.45 [0.44; 0.46] |  | 0.22 [0.21; 0.22] | 0.79 [0.76; 0.82] | 0.21 [0.18; 0.24] |
| 80^th^ percentile | 0.50 [0.47; 0.54] | 0.01 [-0.01; 0.03] | 0.48 [0.46; 0.50] |  | 0.22 [0.20; 0.24] | 0.77 [0.70; 0.84] | 0.23 [0.16; 0.30] |
| 75^th^ percentile | 0.50 [0.49; 0.52] | 0.00 [0.00; 0.00] | 0.50 [0.49; 0.52] |  | 0.22 [0.20; 0.24] | 0.73 [0.66; 0.80] | 0.27 [0.20; 0.34] |

Note: All models adjust for sex and birth year.

**eTable 6.** Sensitivity analyses. Bivariate quantitative genetic sibling models testing alternative shared environmental correlations for the half-siblings. Confidence intervals were derived using the delta method.

| **Maternal half-sibling shared environmental correlation** | **Paternal half-sibling shared environmental correlation** | **Phenotypic**  **correlation** | **Proportion of correlation due to genetic influences** | **Proportion of correlation due to unique environmental influences** |
| --- | --- | --- | --- | --- |
| 0.83 | 0.03 | 0.22 [0.20; 0.24] | 0.97 [0.87; 1.07] | 0.03 [-0.07; 0.13] |
| 0.80 | 0.10 | 0.22 [0.18; 0.25] | 0.98 [0.82; 1.13] | 0.02 [-0.13; 0.18] |
| 0.80 | 0.20 | 0.22 [0.18; 0.25] | 0.98 [0.82; 1.15] | 0.02 [-0.15; 0.18] |
| 0.90 | 0.10 | 0.22 [0.20; 0.24] | 0.99 [0.90; 1.07] | 0.01 [-0.07; 0.10] |
| 0.90 | 0.20 | 0.22 [0.18; 0.25] | 0.98 [0.82; 1.13] | 0.02 [-0.13; 0.18] |
| 1.00 | 0.10 | 0.22 [0.19; 0.25] | 0.97 [0.83; 1.10] | 0.03 [-0.10; 0.17] |
| 1.00 | 0.20 | 0.22 [0.19; 0.25] | 0.97 [0.83; 1.10] | 0.03 [-0.10; 0.17] |

Note: All models adjust for sex and birth year.
